# Ring Opening Polymerization Used for the Production of VOC Free High-Performance Ecofriendly Novel PBZ/PDA/CeO_2_ Nanocomposites

**DOI:** 10.3390/polym15061416

**Published:** 2023-03-13

**Authors:** Mehdi Hatami, Zohreh Seifi Najaf Abadi, Alireza Yousefi, Mohammad Qandalee, Nader Djafarzadeh, Yaser Ghasemi, Ignacio M. López-Coca, Carlos J. Durán-Valle

**Affiliations:** 1 Polymer Research Laboratory, Department of Polymer Science and Engineering, University of Bonab, Bonab 5551761167, Iran; zs70.zohreh@gmail.com; 2 Department of Chemical Engineering, Faculty of Engineering, University of Bonab, Bonab 5551761167, Iran; aliyousefey@gmail.com; 3 Department of Basic Sciences, Garmsar Branch, Islamic Azad University, Garmsar 3581631167, Iran; qandalee@gmail.com; 4 Department of Chemistry, Miyaneh Branch, Islamic Azad University, Miyaneh 1477893855, Iran; n.jafarzadeh@gmail.com; 5 Faculty of Pharmacy, Ramsar Campus, Mazandaran University of Medical Sciences, Ramsar 4691786953, Iran; y.ghasemi@mazums.ac.ir; 6 INTERRA, School of Technology, University of Extremadura, 10003 Cáceres, Spain; iglomar@unex.es; 7 IACYS, Faculty of Sciences, University of Extremadura, Avenida de Elvas, s/n, 06006 Badajoz, Spain

**Keywords:** polydopamine, polybenzoxazine, nanocomposites, synthesis, Mannich reaction

## Abstract

This study analyzed the fabrication and characterization of polybenzoxazine/polydopamine/ceria as tertiary nanocomposites. To this end, a new benzoxazine monomer (MBZ) was fabricated based on the well-known Mannich reaction of naphthalene-1-amine, 2-tert-butylbenzene-1,4-diol and formaldehyde under ultrasonic-assisted process. Polydopamine (PDA) was used as dispersing polymer nanoparticles and surface modifier for CeO_2_ by in-situ polymerization of dopamine with the assistance of ultrasonic waves. Then, nanocomposites (NC)s were manufactured by in-situ route under thermal conditions. The FT-IR and ^1^H-NMR spectra confirmed the preparation of the designed MBZ monomer. The FE-SEM and TEM results showed the morphological aspects of prepared NCs and illustrated the distribution of CeO_2_ NPs in the polymer matrix. The XRD patterns of NCs showed the presence of crystalline phases of nanoscale CeO_2_ in an amorphous matrix. The TGA results reveal that the prepared NCs are classified as thermally stable materials.

## 1. Introduction

Polybenzoxazines (PBZ)s are an innovative category of materials belonging to high-performance engineering thermosets, which do not produce volatile organic compounds (VOC)s during production and remain stable in harsh physical conditions, e.g., heat treatments [1,2,3]. Recently, PBZs exhibited good flame retardancy, easy processability, good flexibility, low dielectric constant, and water absorption properties due to the designed structures [4,5,6,7,8,9,10]. The difference between the phenolic family and the polybenzoxazine macromolecules from structural features is that the phenolic molecules are bonded through the methylene (-CH2-) linkages, while the polybenzoxazine molecules are linked via the formation of benzene rings [11]. Benzoxazine polymers are synthesized using phenols [12,13], primary amines [14,15,16,17], and formaldehyde [18,19,20,21,22,23] via the Mannich reaction under conventional or unconventional methods. Therefore, polybenzoxazines can be synthesized using ring-opening polymerization under thermal conditions with or without catalysts. The excellent properties of benzoxazine macromolecules are related to the Mannich reaction joints and intermolecular hydrogen bonding between an amine and hydroxyl groups. These have led to an increase in the number of polybenzoxazine publications. Wang et al. [24] designed a polymer curing mechanism using three polybenoxazine precursors. Their thermal analysis indicated that the ring-opening process happened powerfully in the ortho-position of aromatic hydroxyl groups of phenols. Li et al. [25] reported on the preparation of polyelectrolyte membranes based on polybenzimidazole and cross-linked polybenzimidazole-polybenzoxazine for application in proton exchange membranes. Kim et al. [26,27] reported on the preparation of polybenzimidazole and polybenzoxazine by the ring-opening process. Moreover, many researchers have reported ring-opening polymerization through the ortho-position of phenols [28,29].

By emerging nanotechnology, the applications of novel instrumental devices have provided a better understanding of the enhancement in properties of nanocomposites (NC)s to the polymer matrices [30,31,32]. From this view, the role of different nanoparticles (NP)s in engineering structures of polymeric NCs has not been denied. Substantial improvements in thermo-mechanical properties have been identified. The active and homogeneous distribution of NPs is quite an impenetrable topic for scientists, which is importantly prejudiced by the intra-particle connections. Therefore, surface modifiers have been used to prevent this problem, placing them in a layer around the nanoparticle [33,34,35,36,37,38,39,40,41].

In this study, cerium oxide (CeO_2_) nanoparticles (NP)s were used as reinforcements due to high thermal resistance. To improve the dispersion and distribution of CeO_2_ NPs in the PBZ matrix, the surface functionalization of NPs was applied. In this case, polydopamine (PDA) was used as a modifier. Dopamine molecules (DA) react with each other in an alkaline solution, producing a biomacromolecule known as PDA, which is a regenerative macromolecule. Dopamine polymerization is performed in different materials, such as unstable metals (palladium, platinum, silver, and statin), metal oxide (aluminum oxide, silicon oxide, and titanium oxide), ceramics, polyurethane, and synthetic polymers (polycarbonate, polystyrene). For the preparation of the benzoxazine monomer, sonication was applied. Moreover, the PBZ/PDA/modified CeO_2_ NCs were prepared by the in situ polymerization method. Fabricated NCs were analyzed using different approaches, such as X-ray diffraction (XRD), Fourier transform infrared (FT-IR) spectroscopy, thermogravimetric analysis (TGA), nuclear magnetic resonance spectroscopy (NMR), transmission electron microscopy (TEM), and field emission scanning electron microscopy (FE-SEM).

## 2. Materials and Methods

### 2.1. Materials and Instruments

Sodium hydroxide, trichlroromethane (CHCl_3_), formaldehyde (37% aqueous solution), tetrahydrofuran (THF), tris(hydroxymethyl)aminomethane (Tris buffer), and dopamine hydrochloride (3-hydroxytyraminium chloride) were purchased from Merck Chemical Company (Darmstadt, Germany). Applied CeO_2_ nanostructures, (particle sizes between 35–45 nm) were provided by Neutrino Chemical Co. (St. Louis, India). Naphtalene-1-amine was purchased from Sigma-Aldrich Company (Now part of Merck KGaA, Darmstadt, Germany). The 2-(tert-butyl)-benzene-1,4-diol was purchased from Elsa Biotechnology Co. (Nanjing, China). FT-IR spectra were recorded at 27 °C in the range of 400–4000 cm^−1^ in a Shimadzu IR affinity-1S spectrometer using KBr pellets. The absorptions due to the vibrational transitions are described in wavenumbers (cm^−1^), and peak strengths were allocated. The ^1^H-NMR spectroscopic technique was conducted with a Bruker Ultrashield 400 MHz NMR spectrometer (Ettlingen, Germany). MBZ was dissolved in an organic solvent (DMSO) at room temperature. The spreading properties characterization of the CeO_2_ into the polymeric medium was studied by application of field emission scanning electron microscopy (FE-SEM, Zeiss-HITACHI (S-4160)-500 nm). The XRD outline was assimilated by using an X’ Pert Pro (Analytical Co., Bangkok, Thailand). The provided graphs were acquired based on 2θ data, in the range of 5–80°, by Cu Kα incident beam (λ = 1.51418 Å). Transmission electron microscopy (TEM) illustrations were provided by a Zeiss-EM10C microscope (Jena, Germany) (acceleration voltage: 100 kV). The NCs samples were crushed and dispersed in water by ultrasonic waves 10 min before the coating process. Thermogravimetric analyses (TGA) of the samples were carried out with a Sanaf-TGA by heating at 10 °C per minute in an ordinary atmosphere from 25 °C to 800 °C.

### 2.2. Monomer Synthesis

For the synthesis of the benzoxazine monomer, 75 mL of CHCl_3_ was poured into a 250 mL flask and kept at a temperature below 5 °C. Then, 9.783 g of 37% formaldehyde solution and 8.62 g of naphthalene-1-amine were added and stirred for two h. Then, 5 g of 2-(tert-butyl)-benzene-1,4-diol was added. The mixture was refluxed for six h, and the reaction temperature was increased slowly to 65 °C. After finishing the reaction, the NaOH solution was added to the mixture for purification. In the next step, the mixture was moved into a 50 mL round-bottom flask, and methanol was added. The obtained material was dissolved in methanol, and then it was poured into an Erlenmeyer and placed in an ice bath for 10 min. Finally, the obtained material was eroded with water, and the benzoxazine monomer was desiccated under vacuum. A similar procedure was reported by Vengatesan [42].

### 2.3. Polymer Synthesis

Two grams of MBZ was dissolved in 5 mL of THF as a solvent under stirring, and the solution was poured into a vessel. Then, the solution was placed under sonication for 15 min at 100 W. Finally, the benzoxazine samples were cured at 210 °C for two h, and samples were cooled at room temperature (27 °C) to obtain polybenzoxazine.

### 2.4. Fabrication of NCs

Two grams of pure CeO_2_ NPs was dehydrated at 120 °C under vacuum conditions for one h. The 0.1 g of dried CeO_2_ NPs was dispersed by sonication process for 10 min in the alkaline buffer solution; at this point, dopamine (0.01 g) was added, and the mixture was stirred for 24 h. Finally, the mixture was separated, and the remained deposition was dried under vacuum conditions. Different amounts of MBZ and adapted CeO_2_ NPs (2, 4, 8, and 10 wt%) were dispersed in 5 mL THF solvent while stirred, and then the mixture was sonicated for 15 min at 100 W. To remove the remaining solvent and perform the curing process, the mixture was dispensed into the mold, and the temperature was adjusted at 210 °C for two h. Finally, NCs with different percentages (NC 2, NC 4, NC 8, and NC 10) were prepared.

## 3. Results

### 3.1. Design and Production of the MBZ Monomer, and the PBZ Polymer

**Scheme 1:**
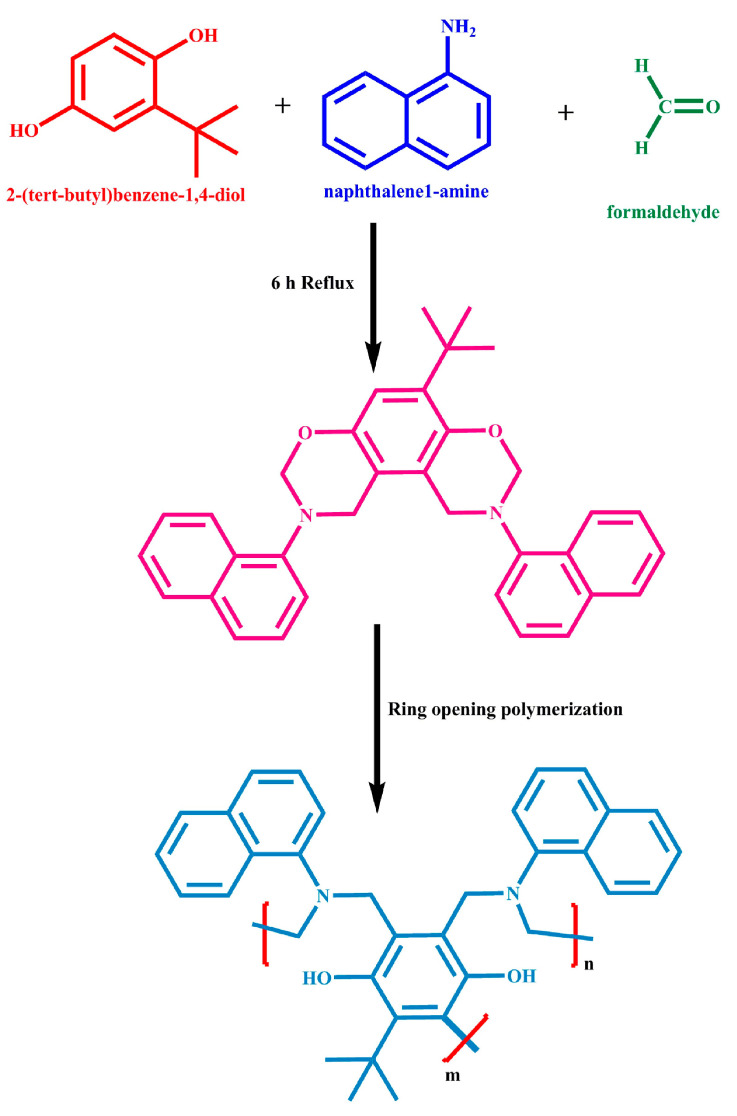
Preparation of MBZ and PBZ.

The schematic illustration of the production procedure for the benzoxazine monomer (MBZ) is displayed in Scheme 1. One of the crucial points in designing a monomer structure is molecular flexibility, which can be improved by creating a novel polymeric structure with excellent and desirable properties [41]. In this study, the benzoxazine monomer was synthesized from the reaction of formaldehyde, naphthalene-1-amine, and the diol functional groups via the well-known Mannich reaction. Through curing by heating at 210 °C, ring-opening polymerization was conducted, and the monomers were jointed to each other. Therefore, the networked system of aromatic rings was observed [34]. Based on the designed structure, the presence of naphthyl groups and aliphatic unsymmetrical units bring more flexibility to the monomer. On the other hand, the presence of aromatic rigid benzene rings strengthens the monomer. By chemical crosslinking reactions of monomers, the final polymer matrix was manufactured. The fabrication of PBZ is also displayed in Scheme 1.

**Figure 1:**
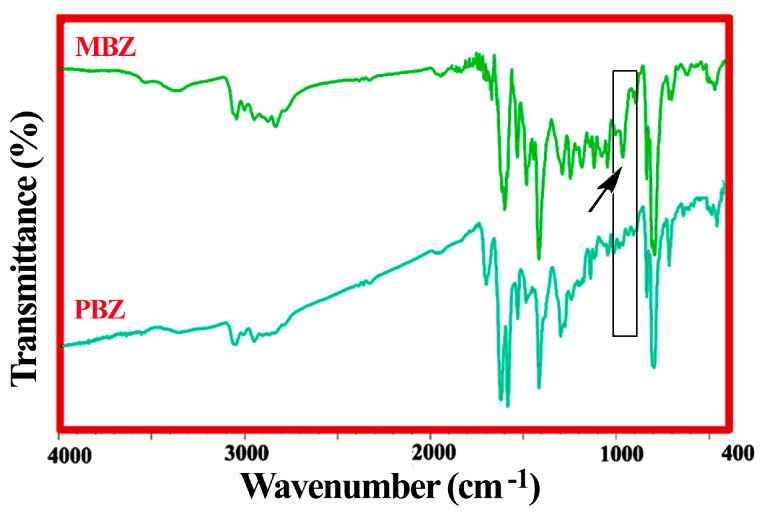
FT-IR spectrum of MBZ monomer and PBZ polymer.

The FT-IR spectra of MBZ and PBZ are shown in Figure 1. In the MBZ spectrum, a band appears at 933 cm^−1^ due to oxazine functionality, which is related to the -N-C-O- stretching mode. This factor was not observed in the spectrum of PBZ, due to the ring-opening polymerization reaction. Furthermore, new bands were observed due to the existence of three aliphatic methyl collections in the benzoxazine structure as symmetric and asymmetric stretching for the MBZ in the range of 2873–2949 cm^−1^, and in the range of 2839–2947 cm^−1^ for PBZ [42].

**Figure 2:**
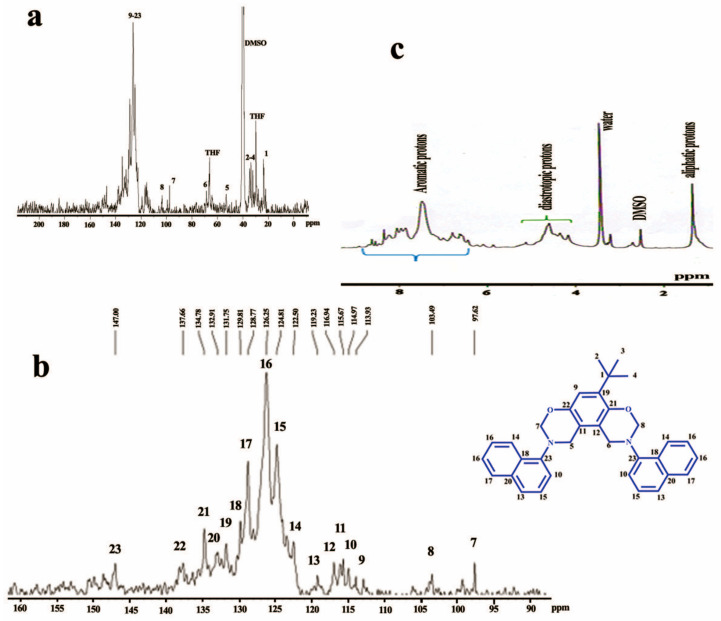
NMR spectra of monomer: (**a**) ^13^C-NMR spectrum; (**b**) ^13^C-NMR spectrum; (**c**) ^1^H-NMR spectrum.

The structure of the MBZ monomer was analyzed by ^1^H-NMR and ^13^C-NMR spectroscopy. The NMR spectra of MBZ are shown in Figure 2a–c. The peak observed at 1.21 ppm corresponds to the hydrogen units of CH_3_ groups (aliphatic protons) in the structure. Aromatic protons resonate in the 6–9 ppm regions. The peaks for oxazine rings are associated with the aliphatic methylene units hanging over the aromatic -CH_2_-N and -O-CH_2_ at 4.45 ppm and 5.15 ppm, respectively (diastotropic protons). The peaks at the ranges of 7.20–8.5 ppm are associated with the hydrogen atoms of the aromatic rings, respectively. The peak at 2.50 ppm was related to the DMSO as a solvent of analysis. Additionally, the peak at 3.40 ppm was concerned with the water molecules. Based on the asymmetry of the structure, presence of diastotropic protons, existence of many aromatic protons by near chemical shifts, and combined aliphatic–aromatic protons, the broadening of the peaks in the range of 4 to 9 ppm is predictable. The spectrum of ^13^C-NMR was provided after the purification with THF. The four aliphatic carbon signals were observed in the 23–34 ppm. In addition, the C-N carbons were observed in the range of 50–70 ppm. The C-O carbons were detected in the range of 97–103 ppm. The seventeen aromatic carbons signals were observed in the range of 113 to 147 ppm. The exact assignments of the carbon signals were illustrated on the molecular structure. Further, due to the purification process and not complete existence of the used solvent, two signals of THF at 27 and 66 were observed. The sharp peak at 39 ppm was matched to the DMSO [43].

### 3.2. Surface Modification of Ceria NPs by Mussel Inspired Sonochemistry

**Scheme 2:**
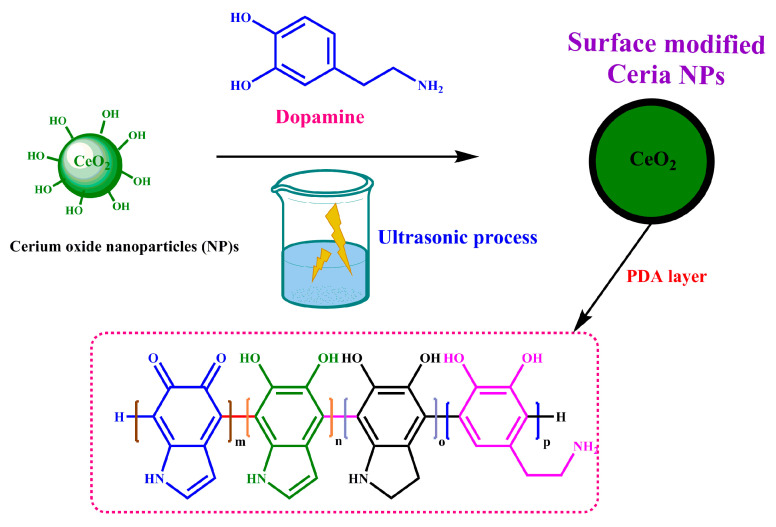
Surface modification process of CeO_2_ NPs by sonication method.

Surface modified cerium oxide NPs with PDA were obtained using ultrasonic waves. The purpose of this reaction is to cover the exterior surface of the nanostructures with aliphatic and aromatic units of PDA as a bio-green structure. The mechanism of surface adjustment of cerium oxide nanostructures with PDA was previously described and is shown in Scheme 2. Ultrasound waves were used in solution media. Some researchers have used this method to modify the surface of different NPs [44,45,46].

**Figure 3:**
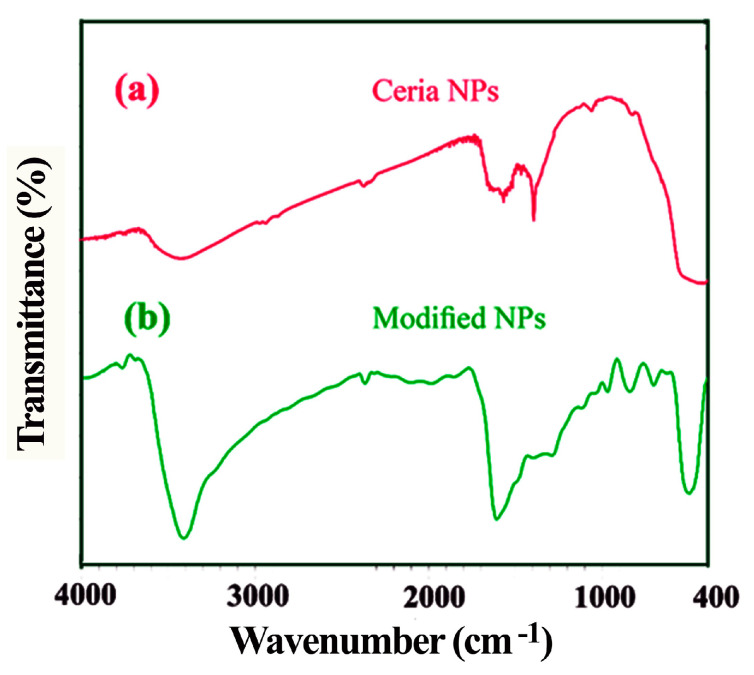
FT-IR spectra of pure CeO_2_ and surface modified CeO_2_ NPs.

**Figure 4:**
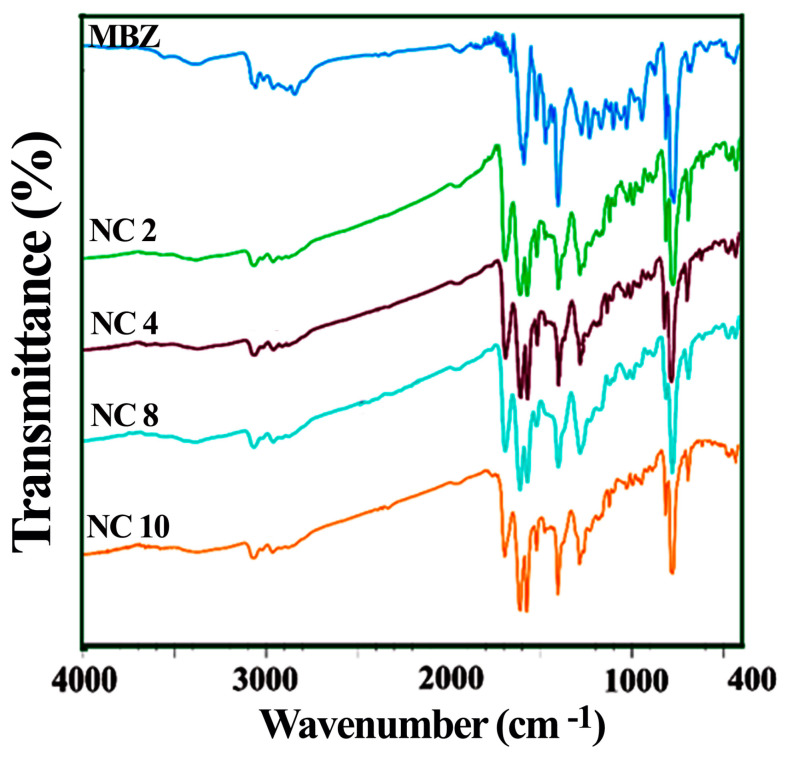
FT-IR spectra of MBZ, PBZ/PDA/CeO_2_ NC 2, PBZ/PDA/CeO_2_ NC 4, PBZ/PDA/CeO_2_ NC 8, and PBZ/PDA/CeO_2_ NC 10.

The FT-IR spectra of the virgin CeO_2_ and surface adjusted CeO_2_ NPs by PDA are displayed in Figure 3. In the FT-IR spectrum of pure cerium oxide, a wide peak at 3421 cm^−1^ can be associated with the absorption of the -OH group of water molecules to cerium oxide. Two peaks were observed with low intensity at 1647 cm^−1^ and high intensity at 464 cm^−1^, which are associated with the vibration bands of the -OH group at the exterior of cerium oxide and the absorption of Ce-O bands, respectively. The spectrum of the cerium oxide NPs modified with PDA is depicted in Figure 4. The vibration peak at 1616 cm^−1^ of the hydroxyl groups was reduced due to the lower amount of adsorbed molecules of water on the surface of the NPs; therefore, the number of hydrogen bonds was smaller.

### 3.3. Nanocomposite Preparation and Characterization

**Scheme 3:**
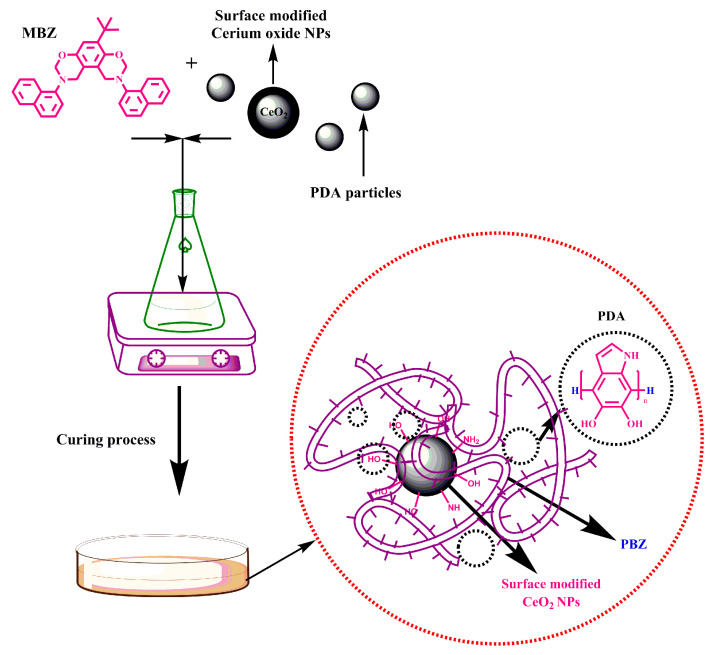
Manufacture of the NCs.

Thermoset polymers such as the PBZs were assigned as the new classes of high-performance polymers. For the preparation of this series of NCs, MBZ and surface-modified NPs were sonicated in the first step. Then, the mixture was poured into a mold, and the polymerization was completed under the thermal process. The schematic illustration of the preparation of NCs is presented in Scheme 3. This nanostructure composite consists of one organic particle, one core-shell organic–inorganic part, and the blended polymer matrix. As a result, two fillers were used in this study. According to the FE-SEM images, the PDA particles, as organic fillers, and surface modified ceria with PDA were assigned. PDA was produced during the surface treatment of NPs. Based on the ultrasonication process, PDA, in many cases, was used to coat the surfaces of CeO2 NPs as nuclear agents. However, in the absence of NPs, the self-PDA particles were also produced.

The FT-IR spectra of CeO_2_ NCs (NC 2, NC 4, NC 8, and NC 10) are presented in Figure 4. According to the results obtained in this analysis, the observed broad peaks between the wavenumbers of 400 to 800 cm^−1^ were associated with the stretching bands of cerium-oxygen connection. For surface-modified CeO_2_ NPs, the observed peak at the ranges of 2920–2890 cm^−1^ and 1500–1400 cm^−1^ correspond to the bending and stretching vibrations of the C-H bands in the methyl and methylene structures, respectively. The PBZ/PDA/CeO_2_ NCs spectrum shows the Ce-O band within the related ranges. Due to the strong vibration bands of the PBZ matrix, the vibration bands of CeO_2_ NPs were not clearly observed. The intensities of NCs peaks vary with the increasing amounts of loaded NPs, which indicate the formation of the modified NPs in the PBZ matrix. Accordingly, these results confirm the successful fabrication of PBZ/PDA/CeO_2_ NCs. Furthermore, the bands at 2949 cm^−1^ and 2831 cm^−1^ were related to the asymmetrical and symmetrical stretching bands of the -CH_2_- group in the aliphatic chain, respectively. The absence of the oxazine band at 933 cm^−1^ indicates the manifestation of ring-opening polymerization in the structure of PBZ/PDA/CeO_2_ NCs. Moreover, the vibration bands in the range of 1390–869 cm^−1^ were related to the vibrations of benzene rings.

**Figure 5:**
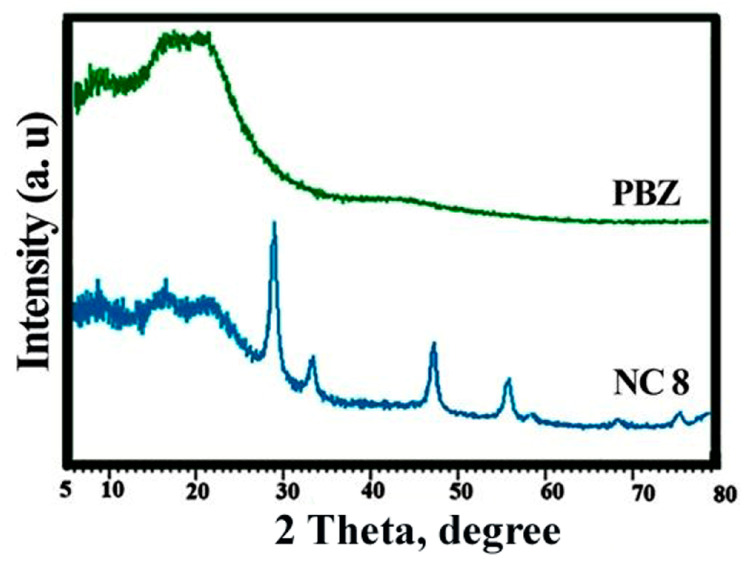
XRD diffraction patterns of PBZ and NC 8.

**Figure 6:**
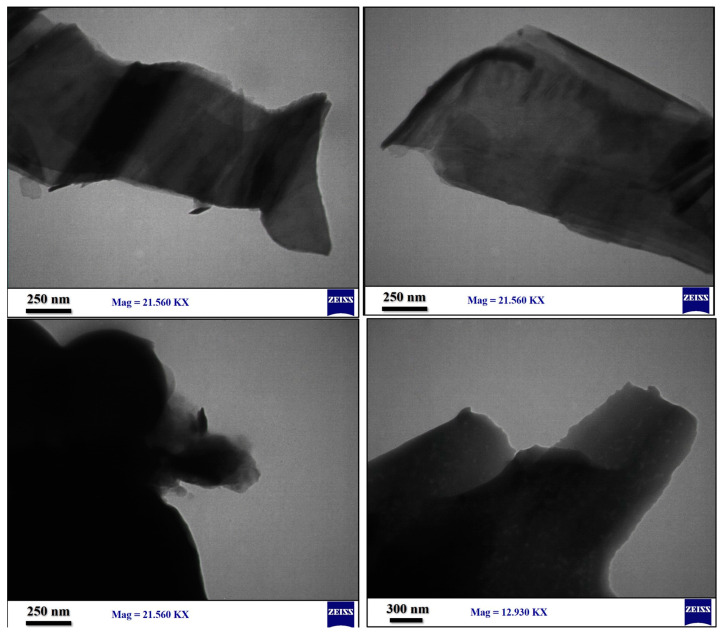
TEM micrographs of PBZ.

XRD is one of the techniques used to study the crystalline properties of materials. The particle size obtained by this technique is similar to that obtained with TEM and SEM. The XRD pattern of pure PBZ and NC 8 (8 wt% of CeO_2_) in the range of 2θ 5–80° is shown in Figure 5. By analyzing the spectrum for the polymer sample (Figure 6), the amorphous phase of the structure can be determined. Based on the XRD pattern for NC 8, a combination peak related to the amorphous phase of the polymer and the crystalline phases of the surface-modified nanostructures can be observed. The broad peak at 2θ = 14–20° and sharp peaks at 2θ = 20–25° are related to the amorphous polymer structure and crystalline phase of nanomaterials, respectively. The crystalline dimension of the nanoscale CeO_2_ was obtained from the Debye–Scherrer equation:

D = 0.9 λ/ß cos θ(1)

where ß is the line width at the half-maximum intensity in radians, λ is the x-ray wavelength, 0.9 is a constant, and θ is the Bragg angle.

The sizes of the NPs were measured at full width and half-maximum. The XRD patterns for the two samples did not show the same patterns in the crystalline regions, although good agreement was obtained for the amorphous peak diffraction pattern of PBZ and NC 8.

**Figure 7:**
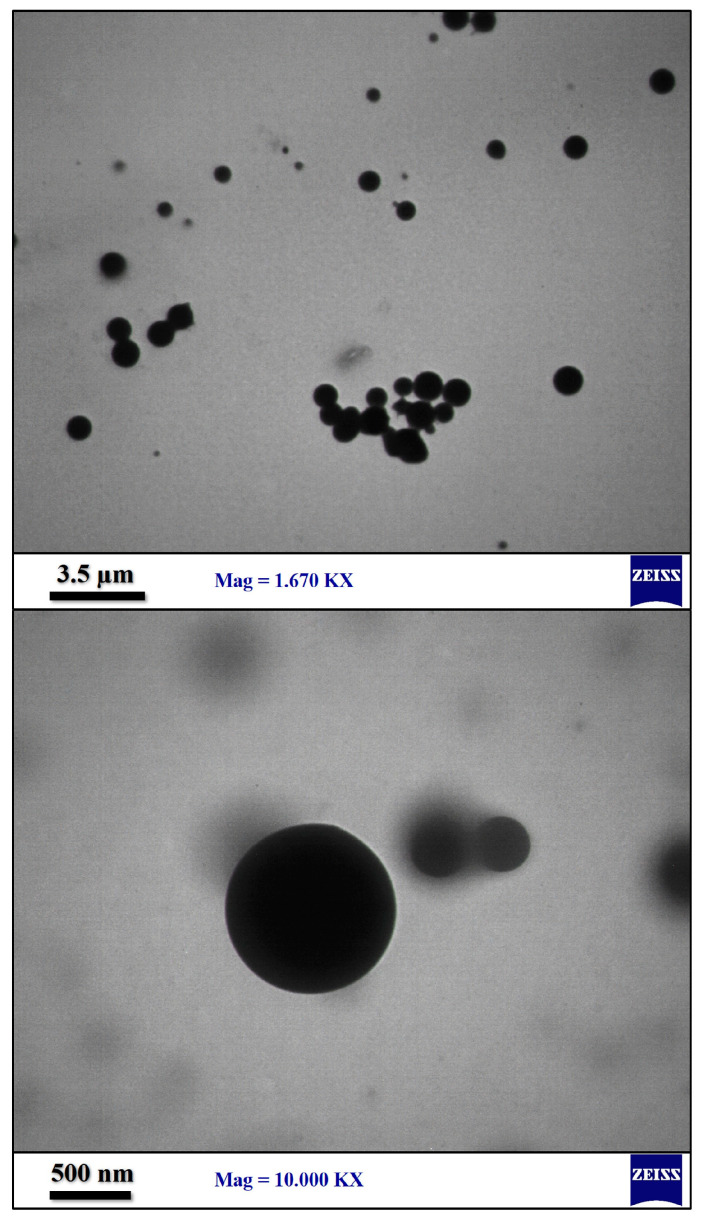
TEM micrographs of surface modified NPs by PDA.

**Figure 8:**
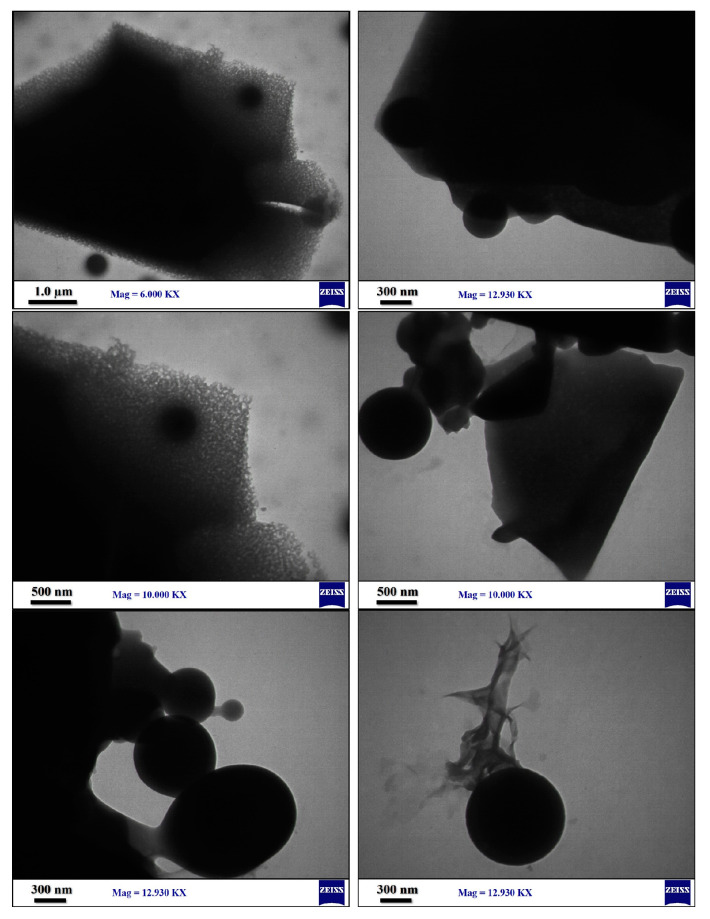
TEM micrographs of NC 8.

TEM analysis is a precision tool to observe the dispersion and dimension of nanomaterials in polymeric NCs. The TEM analysis images for the polymer matrix, hybrid nanoparticles modified by PDA, and NC 8 are illustrated in Figure 6, Figure 7 and Figure 8, respectively. As can be seen, the PBZ matrix shows the bright layer by layer structure. The holes were not observed in the matrix morphology. The smooth structures were detected for the polymer matrix. The morphology of surface-modified NPs is observed in Figure 7. The complete spherical particles for PDA altered NPs were observed in the TEM images. The average particle sizes for modified NPs were measured around 300 nm. Ceria NPs were uniformly coated by PDA, and this organic layer caused better dispersion of NPs in the final polymer matrix in NC structure. The TEM images for NC 8 are presented in Figure 8. The bright layers are associated with the PBZ matrix, and the dark ones in the inner layers represent surface-modified NPs by PDA. These images clearly show the binding of PDA modified CeO_2_ NPs within the matrix. Moreover, it was found that the morphology of the PDA-modified NPs in the NC structure was spherical. The results also show some sufficient attachment of the polymer matrix to spherical particles. It was assumed that some interactions like H-bonding and/or chemical bonds between the backbone chain of PBZ and even the functional units of modified CeO_2_ NPs with the PBZ matrix could be helpful for the construction of NCs. Due to the thick coating of organic PDA on the surface of NPs, the core-shell morphology of the PDA was observed around the exterior of CeO_2_ NPs in the TEM images. Moreover, the formation of the spherical morphology confirms that dopamine was successfully polymerized around the CeO_2_ NPs.

**Figure 9:**
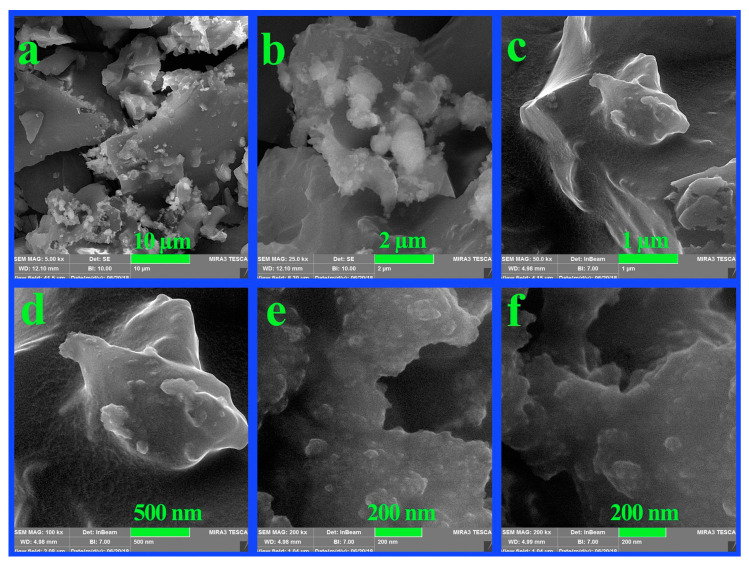
FE-SEM micrographs of NC 2 at different scales.

**Figure 10:**
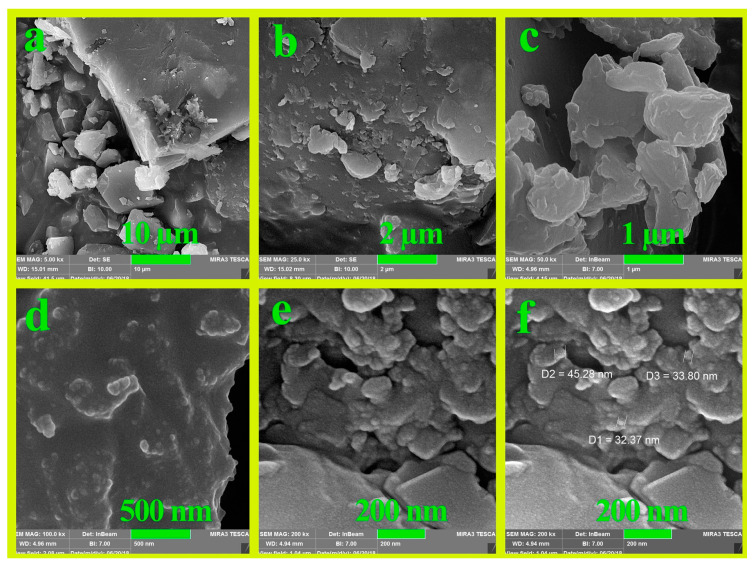
FE-SEM micrographs of NC 4.

**Figure 11:**
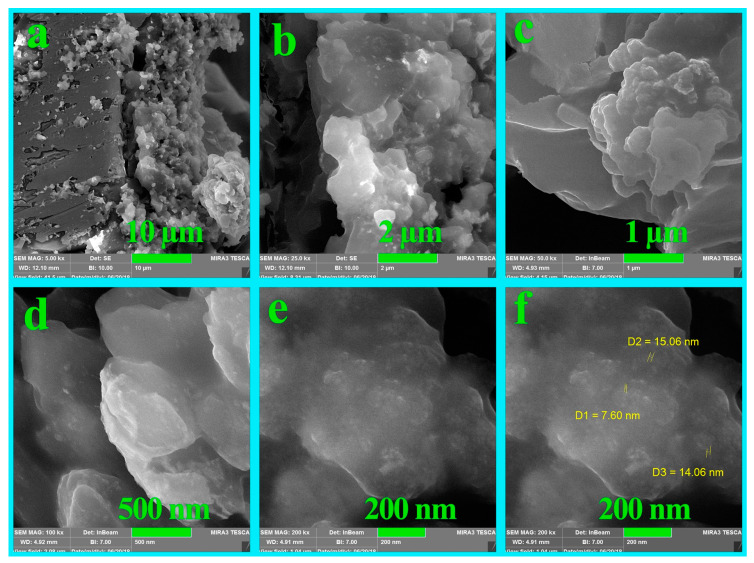
FE-SEM micrographs of NC 8.

The morphology studies of the NCs with different percentages (2, 4, and 8 wt %) as NC2, NC4, and NC 8 were investigated using FE-SEM analysis. The FE-SEM images at a scale of 200 nm to 10 µm were provided for the samples in Figure 9, Figure 10 and Figure 11. The surface morphology of NCs was expressed by adding surface-modified cerium oxide nanoparticles with PDA to the PBZ matrix. In the images of NC 2 (Figure 9), NC 4 (Figure 10), and NC 8 (Figure 11), the surface-modified CeO_2_ NPs are spherical, and by magnifying images at 200 nm, NPs were well distributed in the matrix due to the assistance of ultrasound. Ultrasonic waves caused the homogeneity in morphology. Thus, the functionalization of the surface of the NPs with PDA, used as the binding agent, helped to create the uniform composite material. As a result, the distribution of NPs improved concerning the PDA. NC 2 morphology images (Figure 9) show some sheet structures related to binding due to the functional groups of benzoxazine units and the final formation of a network structure. The network structure can create a good interaction between NPs and the matrix. Figure 10 displays the FE-SEM pictures of the NC 4. The NC 4 displayed slightly asymmetrical morphological characters related to the accumulation of bio-PDA particles. The ceria NPs show an average PDA thickness of 32–45 nm in most cases. In Figure 11, for NC 8, the FE-SEM images show a smutty, but further consistent morphology. The average unit size of the modified NPs in NC 8 was 60 nm.

**Figure 12:**
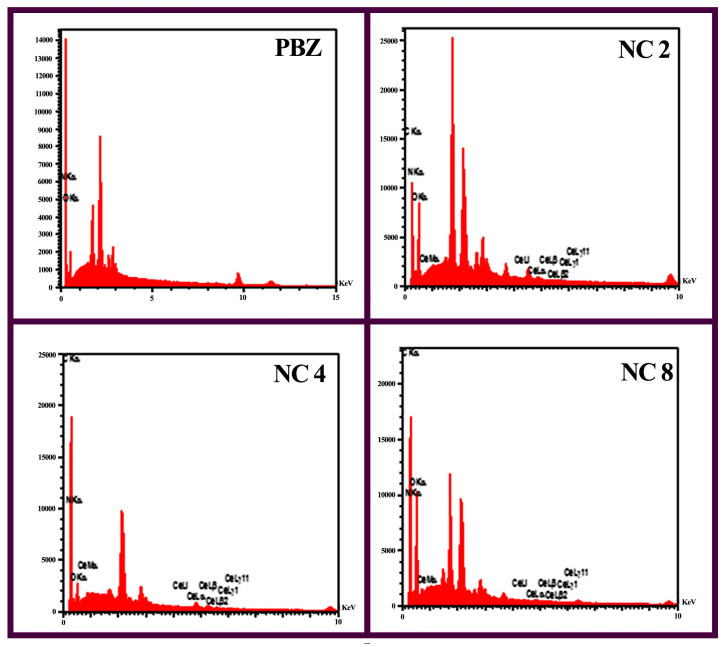
EDS graph of NCs samples.

**Figure 13:**
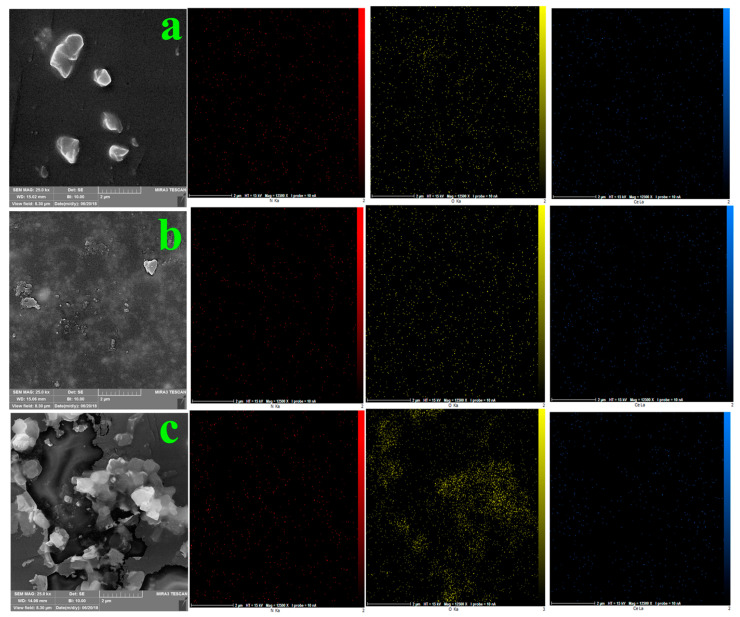
Mapping graphs of NCs samples, (**a**) NC2, (**b**) NC4, and (**c**) NC8. Red for N, yellow for O, and blue for Ce element.

EDS analysis graphs of NCs 2 (b), 4 (c), 8 (d), and PBZ polymer ((a) are shown in Figure 12). The intensity of each spectrum corresponds to the frequency of the element analyzed by EDS. Therefore, the intensity of the lines in the graphs shows the amounts of C, N, O, and the presence of cerium oxide NPs in the samples. Thus, EDS data express increased cerium oxide concentrations in the polymer matrix. Also, a mapping graph of NCs samples is shown in Figure 13.

**Figure 14:**
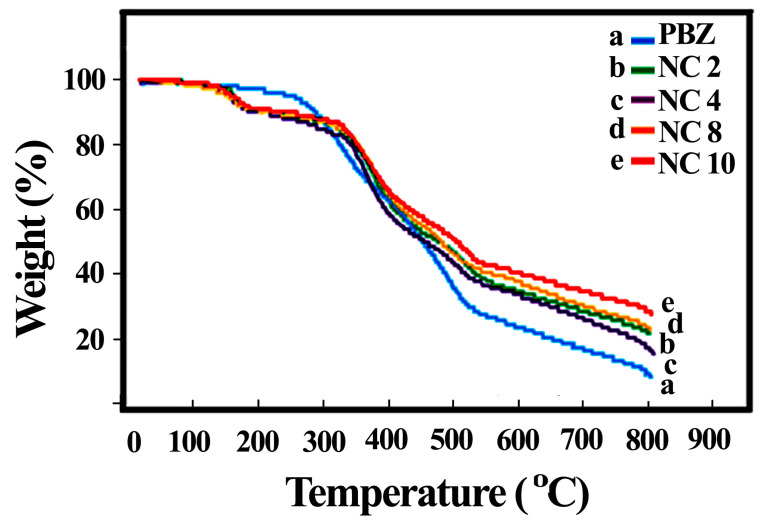
TGA thermograms of NCs.

Figure 14 shows the thermal stability of the PBZ sample and PBZ/PDA/CeO_2_ NCs with different percentages (2, 4, 8, and 10 wt%) at a heating rate of 10 °C min^−1^ in a regular atmosphere from 27 to 800 °C. The thermal stability of PBZ and NCs (2, 4, 8, and 10) obtained from the values of the 5% weight losses (T_5_) and 10% weight losses (T_10_) is reported in Table 1. The high thermal resistances of NCs are related to the good thermal strength of the network structure of the matrix and the inorganic nature of CeO_2_ nanostructures. For this reason, the presence of PDA modified CeO_2_ NPs in a polymer matrix creates a networked aromatic structure. In fact, the hydroxyl groups and aromatic nature of PDA increase the thermal stability and flame retardancy of NCs. The remaining char yield (C.Y.) was related to the carbonization of the samples in an air atmosphere at 800 °C. The limiting oxygen index (LOI) can be determined by analyzing the C.Y. values of samples. LOI is obtained using the Van Krevelen equation [47]:

LOI = 17.5 + 0.4 C.Y.(2)

where C.Y. is defined as the char yield of materials.

Then, by determining LOI, the specific heat of combustion (∆H_comb_) can be obtained from the Johnson equation:

LOI = 8000/∆H_comb_ (3)

The LOI and ∆H_comb_ values are reported in Table 1.

## 4. Conclusions

A new benzoxazine monomer (MBZ) was prepared from the reactions of three species (naphtalene1-amine, formaldehyde, and 2-tert-butylbenzene-1,4-diol) through Mannich reaction. PBZ/PDA/CeO_2_ NCs with different concentrations of CeO_2_ nanostructures were manufactured using the sonochemical technique. The prepared samples were studied by FT-IR, ^1^H-NMR, FE-SEM, TEM, XRD, and TGA. Using TEM and FE-SEM analyses, the homogeneous distribution of cerium oxide NPs in a polymer matrix was observed. PBZ offered amorphous construction, and NCs revealed the crystal-like structure of nanoscale ceria and the amorphous phase of the matrix. By increasing the amount of cerium oxide, NPs improved, in most cases, the thermal stability parameters of NCs, which is due to the characteristics of cerium oxide NPs. 

## Figures and Tables

**Table 1:** Thermal properties of the PBZ, and PBZ/CeO_2_ NCs.

Sample	T_5_ (°C) ^a^	T_10_ (°C) ^b^	Char Yield (%) ^c^	LOI ^d^	∆H_comb_
PBZ	243	294	9	17.53	456.36
NC 2	164	209	22	17.58	455.06
NC 4	155	195	16	17.56	455.58
NC 8	154	210	23	17.59	454.80
NC 10	163	221	28	17.61	454.28

^a^ Temperature at which 5% weight loss was recorded by TGA at heating rate of 10 °C/min under air atmosphere. ^b^ Temperature at which 10% weight loss was recorded by TGA at heating rate of 10 °C/min under air atmosphere. ^c^ Weight percentage of material left undecomposed after TGA analysis at a temperature of 800 °C under air atmosphere. ^d^ Limiting oxygen index (LOI) determined by char yield at 800 °C.
